# Scutellarin Alleviates Cuprizone-Induced Demyelination by Improving Mitochondrial Dysfunction, Reducing Lipid Oxidation and Inhibiting the p38 MAPK Pathway

**DOI:** 10.3390/antiox14060723

**Published:** 2025-06-12

**Authors:** Qiting Zhao, Yantuanjin Ma, Shufen Wang

**Affiliations:** Yunnan Key Laboratory of Breast Cancer Precision Medicine, Academy of Biomedical Engineering, Kunming Medical University, Kunming 650500, China; zqt940601@126.com (Q.Z.); 20190329@kmmu.edu.cn (Y.M.)

**Keywords:** scutellarin, cuprizone, microglia, mitochondrial dysfunction, lipid oxidation, demyelination

## Abstract

The occurrence of demyelination in the central nervous system (CNS) causes neurodegenerative lesions. The occurrence and development of demyelination involve multiple pathological mechanisms, including the generation of reactive oxygen species (ROS) caused by mitochondrial dysfunction in microglia and subsequent neuroinflammation. Scutellarin is a natural flavonoid drug with significant neuroprotective effects, including antioxidant, anti-inflammatory, and anti-apoptotic properties, and is widely used in the treatment of neurological diseases. However, the protective effects and mechanisms of scutellarin on demyelination have not yet been elucidated. This study aims to investigate the neuroprotective effects of scutellarin on demyelination and its underlying molecular mechanisms. Our results showed that treatment with scutellarin significantly alleviated Cuprizone-induced myelin damage, neuronal apoptosis, and neurological deficits in mice. In in vitro experiments, scutellarin significantly reduced Cuprizone–copper-induced pro-inflammatory microglia formation and inhibited the secretion of TNF-α, thereby reducing myelin cell damage. Mechanism studies revealed that scutellarin inhibited the secretion of TNF-α by microglia and alleviated myelin cell damage by reducing the excessive production of mitochondrial reactive oxygen species (Mito-ROS), reactive oxygen species (ROS), and malondialdehyde (MDA) induced by Cuprizone–copper in microglia. Finally, scutellarin improved mitochondrial dysfunction in microglia and significantly alleviated myelin cell damage by inhibiting the expression of p38MAPK. In conclusion, our findings demonstrate that scutellarin exerts significant neuroprotective effects on Cuprizone-induced mice by improving mitochondrial dysfunction in microglia, thereby reducing inflammatory responses. This effect is closely associated with the inhibition of the p38MAPK pathway.

## 1. Introduction

Myelin is a structurally complex membrane that tightly wraps around the periphery of nerve fibers to ensure effective transmission of nerve impulses [1,2,3]. When the integrity of myelin is compromised, it not only hinders the transmission of nerve impulses, but also triggers irreversible neurological dysfunction [4,5]. More common causative factors in clinical practice include congenital disorders of myelin development [6], pathogenic infections [7], metabolic abnormalities [8], and dysregulation of the immune system [9,10,11]. Therefore, revealing the underlying mechanisms of demyelinating-related diseases and developing new therapeutic modalities are urgent needs for preventing demyelination.

The Cuprizone (CPZ) mouse model is currently widely used as an animal model for studying demyelination of the central nervous system [12,13]. Feeding mice with feed or drinking water containing 0.2% CPZ can induce extensive demyelination in the central nervous system of mice. In the CPZ-induced toxic demyelination model in mice, cellular damage of oligodendrocytes can be widely observed. In addition, in demyelinated areas, the activation of pro-inflammatory microglia and the secretion of the inflammatory cytokine TNF-α can cause demyelination [14,15,16,17]. Myelin-associated glycoprotein (MAG) and myelin basic protein (MBP) are reduced [18,19]. Furthermore, it can lead to dysregulation of mitochondrial function in cells, followed by an increase in intracellular ROS production, thereby aggravating oxidative stress [20,21]. CPZ is a copper ion chelator that can form metal complexes with copper [21]. Studies have shown that the demyelinating effect of CPZ is not due to copper chelation, but rather to acute copper overdose caused by the formation of metal complexes with copper [22]. However, the study only focused on phenotype by using other copper chelators for treatment, and the more detailed mechanisms have not been thoroughly explored.

Copper is a metal pollutant, and environmental studies suggest that copper may increase the risk of demyelination [23,24,25,26,27]. In addition, studies have shown that in demyelinating diseases induced by N,N-diethyldithiocarbamate, copper accumulation precedes inflammation and myelin lesions [28,29,30,31,32,33,34]. Clinical studies have shown that copper levels in cerebrospinal fluid (CSF) and blood are significantly elevated in multiple sclerosis (MS) patients [35,36,37,38,39]. Furthermore, elevated copper levels in cerebrospinal fluid have been detected in Skogholt disease, a neurological disease characterized by myelin damage [40,41]. Additionally, demyelinating lesions have been observed in patients with Wilson’s disease, a genetic disorder in which the body cannot metabolize copper [42,43,44,45,46,47].

Mitochondria are one of the main sites of copper utilization in cells. In the case of excessively high intracellular copper levels, copper is transported to the mitochondrial matrix for storage to prevent copper overload, which may also cause damage to mitochondria [48,49]. So, does CPZ cause demyelination in mice by increasing mitochondrial copper levels in cells, leading to mitochondrial damage?

Microglia are resident immune cells of the central nervous system, and the inflammatory factors they produce can cause oligodendrocyte damage, leading to demyelination and ultimately neurological dysfunction. Although microglia can produce neurotrophic factors to repair the central nervous system, they can also participate in neuroinflammation and neurodegeneration in demyelinating diseases by releasing inflammatory mediators. Microglial activation occurs before the onset of demyelinating related diseases, and inhibiting this early activation can hinder the development of CNS inflammatory lesions to a certain extent [50,51,52,53]. CPZ exposure can affect gene expression in the hippocampus of mice, with microglia being the most susceptible. CPZ exposure can activate microglia to produce pro-inflammatory factors that damage oligodendrocytes and neurons [21]. However, the role and pathogenic mechanisms of microglia in this process remain uncertain.

There are many clinical treatments for demyelinating related diseases, which can have significant short-term efficacy, but long-term treatment is prone to recurrence, expensive, difficult to cure fundamentally, and has certain side effects [54]. Therefore, the development of new effective drugs with fewer side effects remains a research hotspot. Scutellarin (SCU) is a flavonoid substance derived from the herb *Erigeron breviscapus* Hand-Mazz, which is recorded in the Chinese herbal medicine classic “Diannan Bencao”. Scutellarin can increase the permeability of the blood–brain barrier (BBB) and has significant neuroprotective effects such as anti-inflammatory, antioxidant, and anti-apoptotic properties [55,56,57,58,59,60,61]. Previous studies have shown that scutellarin has therapeutic effects on demyelinating related diseases in different cell and animal experimental models [62,63,64]. However, so far, it is unclear how this drug treats neuroinflammation induced by microglia with a Pro-inflammatory cell phenotype. Therefore, inhibiting the abnormal activation of microglia by scutellarin is crucial for understanding the pathogenesis and repair treatment of demyelinating-related diseases. However, the underlying molecular mechanisms of scutellarin in the treatment of demyelinating-related diseases remain to be elucidated.

Therefore, in this study, we attempted to further investigate the potential neuroprotective effects of scutellarin on a CPZ-induced demyelination model in mice. In addition, we investigated whether the neuroprotective mechanism of scutellarin is related to improving mitochondrial dysfunction in microglia. This study provides a potential therapeutic strategy for the treatment of demyelinating-related diseases.

## 2. Materials and Methods

### 2.1. Experimental Animals

The 8-week-old adult male (20–25 g) C57BL/6 mice used in this study were purchased from Beijing Sibefu Biotechnology Co., Ltd. (Beijing, China) and housed by the Laboratory Animal Department of Kunming Medical University. The ambient temperature of the animal facility was maintained at around 25 ± 3 °C, with a 12:12 h light–dark cycle, in a quiet environment to avoid noise and strong light stimulation. All animal experiments were approved by the Kunming Medical University and the Animal Ethics Committee of Kunming Medical University (Approval No.: KMMU20241583, 13 June 2024), and were conducted in accordance with the relevant guidelines issued by the International Council for Laboratory Animal Science and the ethical policies for animal use.

### 2.2. Experiment Groups and Drug Administration

Twelve-week-old mice were randomly divided into four groups: a normal diet group (*n* = 10), a CPZ-fed group (*n* = 10), a CPZ-fed plus saline injection group (*n* = 10), and a CPZ-fed plus scutellarin treatment group (*n* = 10), with five mice per cage. Mice in the CPZ-fed and CPZ-fed plus saline injection groups were given 8 g of cylindrical feed containing 0.2% CPZ (Merck, Burlington, MA, USA) daily. The cylindrical feed containing Cuprizone was prepared and stored at 4 °C in the dark. The CPZ feed was replaced daily for a total of 3 weeks. The mice were weighed daily. And after CPZ feed for 1 week, they were treated with intraperitoneal injections of 100 mg/kg.d scutellarin (MCE, Dallas, TX, USA) 2 weeks, with an equal volume of saline as a control, along with CPZ feeding. Scutellarin was given daily for the 2 weeks. After three weeks of CPZ treatment with or without scutellarin, the mice were evaluated with behavioral testing and then sacrificed and tissue collected for immunofluorescence analysis, qPCR analysis, and Western blot analysis. The neuronal apoptosis (Nissl staining kit; Beyotime, Shanghai, China) was evaluated according to the manufacturer’s instructions. The experiment was independently repeated three times.

### 2.3. Cell Culture and Treatment

BV2 cells (immortalized cell lines of microglia) were kindly provided by Dr. Qiuye Jia (Kunming Medical University, Kunming, China). MO3.13 cells (immortalized cell lines of oligodendrocytes) were purchased Warner Bio (Wuhan, China). All cell lines (BV2, MO3.13) were maintained in DMEM high glucose supplemented (Gibco, Waltham, MA USA) with 10% fetal bovine serum (FBS) (Opcel, Hohhot, China), and incubated at 37 °C in air containing 5% CO_2_. A total of 6 × 10^5^ cells in 2 mL DMEM containing 10% FBS were plated per well into six-well plates. After 1 day, the cells were pretreated with p38 inhibitor (10 μM, SB203580, MCE, USA), Ferrostatin-1 (1 μM, Fer-1, MCE, USA), Ammonium tetrathiomolybdateat (10 μM, ATTM, MCE, USA) and Scutellarin (20 μM, Scu, MCE, USA) overnight before Cuprizone (MCE) exposure, respectively.

After 24 h, all cell lines (BV2, MO3.13) were collected for cell viability analyses. The cell viability (CCK-8 assay kit, Beyotime, China) kits were used according to the manufacturer’s instructions. In addition, BV2 cell supernatants were collected for co-culture and ELISA. BV2 cells were collected for qPCR and Western blot. The experiment was performed in triplicates and repeated three times.

### 2.4. Y-Maze

Mice were individually placed in the center of the Y-maze and allowed to adapt to the environment for 5 min. At the start of the experiment, the mice were again placed in the center of the maze. The arm first chosen by the mice was recorded, as well as the number of entries and duration spent in each arm. The total duration of the experiment was 5 min. Each mouse underwent three independent repeated trials.

### 2.5. Open Field Test

Before the experiment, the mice were allowed to adapt to the laboratory environment for 30 min. At the start of the experiment, the mice were placed in the center of the open field apparatus. The behavior of the animals in the open field was then recorded, including the time spent in the center and periphery of the field, as well as the distance moved. The observation period was 5 min. Each mouse underwent three independent repeated trials.

### 2.6. Western Blot Analysis

Tissues or cells were lysed with 200 μL RIPA buffer (Beyotime, Shanghai, China) containing 1% PMSF (Beyotime, Shanghai, China) and 1% phosphoprotein inhibitor. The proteins were then denatured at 100 °C for 10 min. Protein concentration was determined using a BCA Assay (Beyotime, Shanghai, China). Proteins were separated by SDS-PAGE (Yamei, Shanghai, China) and transferred to PVDF membranes (Merck, MA, USA). The membranes were then incubated with appropriate primary and secondary antibodies, including JNK Antibody (1:1000, #9252, MW (kDa): 46,54, Cell Signaling Technology, Danvers, MA, USA), p-JNK Antibody (1:1000, #9251, MW (kDa): 46,54, CST), p38 MAPK Antibody (1:1000, #9212, MW (kDa): 40, CST), p-p38 MAPK Antibody (1:1000, #9211, MW (kDa): 40, CST), ERK1/2 Antibody (1:1000, #9102, MW (kDa): 42,44, CST), p-ERK1/2 Antibody (1:1000, #9101, MW (kDa): 42,44, CST), MBP Antibody (1:1000, #78896, MW (kDa): 18, CST, Danvers, MA, USA), MAG Antibody (1:1000, #9043, MW (kDa): 69, CST), and β-actin Antibody (1:1000, #BA2305, MW (kDa): 45, BOSTER, Wuhan, China). Three independent experiments were performed for statistical analyses.

### 2.7. Immunofluorescence Staining

Frozen sections of fresh mouse brain tissue were prepared. The thickness of the sections was 10 μm. The sections were subjected to antigen retrieval using citrate. The sections were then blocked with 5% BSA for 30 min. The sections were incubated with primary antibodies overnight at 4 °C. The sections were then washed with PBST. The sections were incubated with secondary antibodies in the dark for 1 h. The sections were then mounted with an anti-fade mounting medium containing DAPI. The sections were observed and photographed under a fluorescence microscope. A holographic scanner (3DHISTECH, Budapest, Hungary) was used to take pictures. Data processing was performed using ImageJ 1.52n software. Data analysis was performed using GraphPad Prism 8. Data were analyzed using *t*-tests (and non-parametric tests). Iba-1 Antibody was used (1:1000, Servicebio, Wuhan, China). Other primary antibodies were the same as above.

### 2.8. Real-Time Quantitative Polymerase Chain Reaction (RT-qPCR)

Total RNA was extracted from cells using RNAiso Plus (TaKaRa, Dalian, China). Reverse transcription of total RNA was performed using PrimeScript RT Master Mix (TaKaRa, Dalian, China). After reverse transcription, RT-qPCR analysis of mRNA levels was performed using SYBR Green Real-time PCR Master Mix kit (TaKaRa, Dalian, China) under the following conditions: initial pre-incubation at 95 °C for 30 s, followed by 39 cycles at 95 °C for 5 s and 60 °C for 30 s. Gene levels were calculated by the 2^−ΔΔCt^ method. Three independent experiments were performed for statistical analysis. The sequences of primers are listed in Table S1.

### 2.9. Detection of Intracellular Copper Content

After collecting tissue or cell samples, the copper uptake levels of the cells were determined using a copper assay kit (Solarbio, Beijing, China) according to the manufacturer’s instructions. Three independent experiments were performed for statistical analysis.

### 2.10. Transmission Electron Microscopy

The morphological changes of mitochondria in the BV2 cells were observed using transmission electron microscopy (TEM) (JEM-1400, JEOL Ltd., Tokyo, Japan).

### 2.11. Mitochondria Isolation

After collecting tissue or cell samples, mitochondria were extracted from cells/tissues using a mitochondrial isolation kit (Beyotime, Shanghai, China) according to the manufacturer’s instructions.

### 2.12. ELISA

The concentration of TNF-α in the cell culture supernatant was measured using a TNF-α ELISA kit (BOSTER, Wuhan, China) according to the manufacturer’s instructions. Three independent experiments were performed for statistical analysis.

### 2.13. Mitochondrial Function Testing

After treating cells with Cuprizone, changes in mitochondrial function were detected. Briefly, the levels of reactive oxygen species (ROS), mitochondrial membrane potential, mitochondrial ROS, lipid peroxidation, and ATP in cells were measured using the DCFH-DA Kit (Beyotime, Shanghai, China), JC-1 Kit (Beyotime), MitoSOX Kit (Beyotime), MDA Kit (Beyotime), and ATP Kit (Beyotime), respectively, according to the manufacturer’s instructions. The levels of ROS, mitochondrial membrane potential, and mitochondrial ROS were detected by flow cytometry. The levels of MDA and ATP were detected by a multi-functional microplate reader(Molecular Devices, Sunnyvale, CA, USA).

### 2.14. Statistical Analyses

All experiments were independently repeated three times. Statistical analyses and graph generation were performed using GraphPad Prism 8 software. Data were analyzed with *t*-tests (and non-parametric tests) and expressed as mean ± standard error of the mean (mean ± s.e.m). For comparing more than two groups, an ANOVA test is used (one-way or two-way) followed by *t*-tests (and non-parametric tests) to determine which specific groups differ significantly. The results of ANOVA tests are listed in Table S2. Images were adjusted using Photoshop and Adobe Illustrator. * indicates *p* < 0.05 was considered statistically significant. **, *** and **** indicate *p* < 0.01, *p* < 0.001 and *p* < 0.0001 were considered highly statistically significant, respectively.

## 3. Result

### 3.1. In Vivo Experiments Demonstrate That Scutellarin Alleviates Cuprizone-Induced Demyelination in Mice

**Figure 1:**
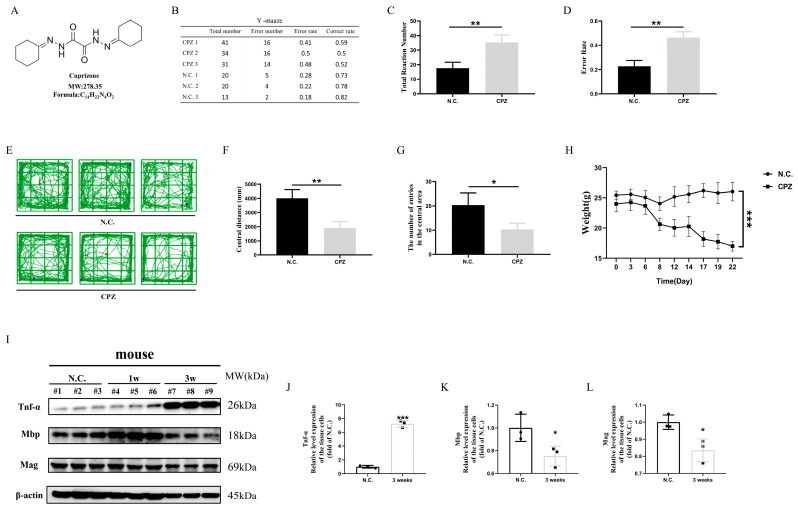
Cuprizone induces demyelination, brain damage, and neurological deficits in mice. (**A**) Chemical information of Cuprizone. (**B**–**G**) Effects of Cuprizone treatment on neurological function in mice. (**H**) Effects of Cuprizone treatment on body weight in mice. (**I**–**L**) Western blotting analysis of TNF-α, MBP, and MAG protein expression in brain tissue of Cuprizone-treated mice. * *p* < 0.05, ** *p* < 0.01, *** *p* < 0.001. Three mice per group.

To evaluate the therapeutic efficacy of scutellarin, we first established a Cuprizone-induced demyelination model in mice (Figure 1A). In our experiments, we initially investigated the impact of Cuprizone on murine neurocognitive functions by observing behavioral changes. In the Y-maze test, Cuprizone-fed mice exhibited significantly higher error rates (re-entering the same arm as the previous two entries) compared to the normal diet group, indicating impaired learning and memory capabilities. Additionally, Cuprizone-fed mice showed significantly more total arm entries in the Y-maze test than the control group, suggesting increased anxiety and irritability after Cuprizone administration (Figure 1B–D). In the open field test, Cuprizone-fed mice demonstrated significantly reduced central zone entries and total movement distance compared to the normal diet group, indicating decreased exploratory behavior and cognitive function under Cuprizone exposure (Figure 1E–G). Furthermore, during the Cuprizone feeding period, mice exhibited a continuous weight loss starting one week post-administration (Figure 1H).

Subsequently, we examined histopathological changes in the murine brain tissues. Western blotting analysis revealed significantly elevated protein expression of the pro-inflammatory cytokine TNF-α (a critical factor in multiple sclerosis with myelinolytic effects [21]) and reduced expression of myelin-associated proteins MBP and MAG in Cuprizone-fed mice compared to controls (Figure 1I–L). Immunofluorescence analysis showed decreased MBP fluorescence intensity in the brains of Cuprizone-treated mice compared to healthy controls (Figure S1A), indicating oligodendrocyte damage and subsequent demyelination. Additionally, enhanced Iba1 fluorescence intensity and morphological changes in microglia suggested activation of resting microglia (Figure S1B). Collectively, these findings confirmed successful establishment of the Cuprizone-induced demyelination model.

**Figure 2:**
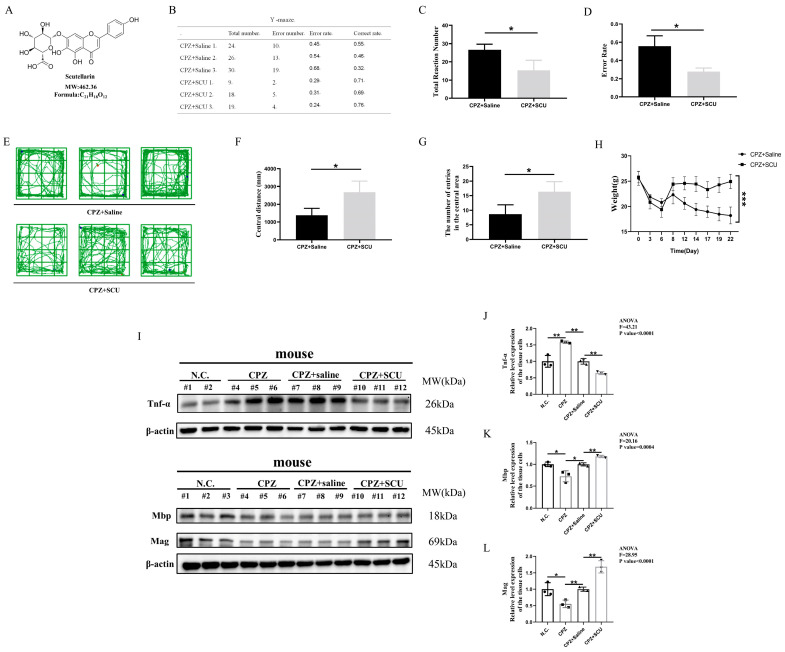
Scutellarin alleviates Cuprizone-induced demyelination, brain damage, and neurological deficits in mice. (**A**) Chemical information of Scutellarin. (**B**–**G**) Effects of Scutellarin treatment on neurological function in Cuprizone-treated mice. (**H**) Effects of Scutellarin treatment on body weight in Cuprizone-treated mice. (**I**–**L**) Western blotting analysis of TNF-α, MBP, and MAG protein expression in brain tissue of Scutellarin-treated mice. * *p* < 0.05, ** *p* < 0.01, *** *p* < 0.001. Three mouse per group.

And after CPZ feed for 1 week, they were treated with intraperitoneal injections of scutellarin for 2 weeks, along with CPZ feeding. Scutellarin was given daily for the 2 weeks (Figure 2A). We assessed the therapeutic effects of scutellarin using neurobehavioral tests. In the Y-maze test, scutellarin-treated mice showed significantly reduced error rates, indicating restored learning and memory capabilities. Furthermore, scutellarin-treated mice exhibited significantly fewer total arm entries than Cuprizone-fed mice, suggesting alleviated anxiety and irritability (Figure 2B–D). In the open field test, scutellarin-treated mice demonstrated increased central zone entries and movement distance, indicating restored exploratory behavior and cognitive function (Figure 2E–G). Additionally, mice started to regain weight after scutellarin administration (Figure 2H). Western blotting analysis revealed significantly reduced TNF-α protein expression and restored MBP and MAG levels in scutellarin-treated mice compared to Cuprizone-fed controls (Figure 2I–L). Immunofluorescence results showed that MBP fluorescence intensity was higher in the brains of scutellarin-treated mice than in scutellarin-untreated mice (Figure S1C), suggesting that scutellarin has a protective effect against with myelin damage indicating remission of myelin damage.

Collectively, these findings demonstrate the significant therapeutic efficacy of scutellarin in Cuprizone-induced demyelination.

### 3.2. In Vitro Experiments Reveal That Scutellarin Alleviates Myelin Cell Damage by Inhibiting Cuprizone-Induced Pro-Inflammatory Microglial Activation

**Figure 3:**
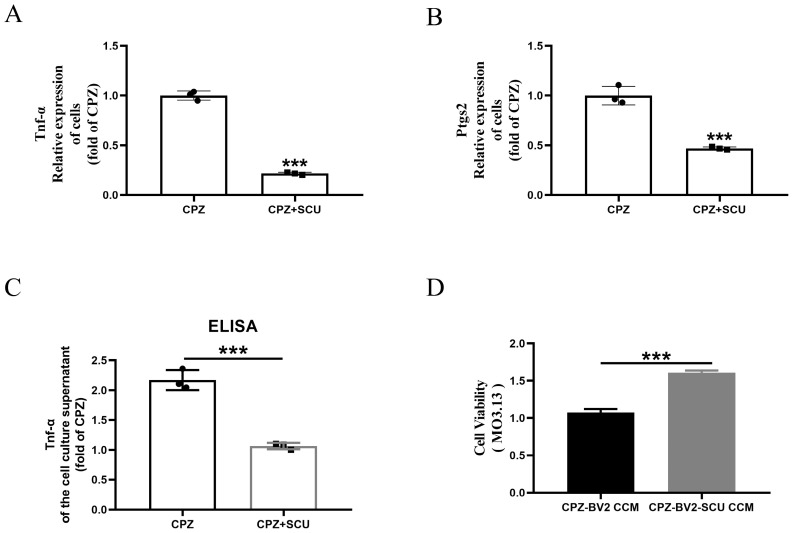
In vitro, Scutellarin alleviates myelin cell damage by inhibiting Cuprizone-induced pro-inflammatory microglia activation. (**A**–**C**) Scutellarin treatment inhibits the formation of pro-inflammatory phenotypes in microglia. (**D**) Effects of conditioned medium from Scutellarin-treated microglia on cell viability in MO3.13 cells. *** *p* < 0.001. All experiments were independently repeated three times.

In vitro, scutellarin (20 μM/mL) treatment of BV2 cells significantly inhibited the gene expression of pro-inflammatory cytokines *TNF-α* and *Ptgs2* (Figure 3A,B). ELISA analysis confirmed that scutellarin effectively reduced TNF-α secretion into the culture medium by BV2 cells (Figure 3C). Finally, the MO3.13 cells were co-cultured with media from BV2 cells treated with Cuprizone alone or with Cuprizone + scutellarin for 24 h, which increased cell viability in myelin cells (Figure 3D).

### 3.3. Cuprizone Forms Complexes with Copper, Inducing Cellular Copper Intoxication and Promoting Pro-Inflammatory Microglial Activation

Recent studies suggest that Cuprizone’s demyelinating effects are not due to copper deficiency but are consistent with the acute toxicity of CPZ–copper(II) complexes. However, these studies have primarily focused on phenotypic outcomes without delving into detailed cellular and molecular mechanisms [22].

**Figure 4:**
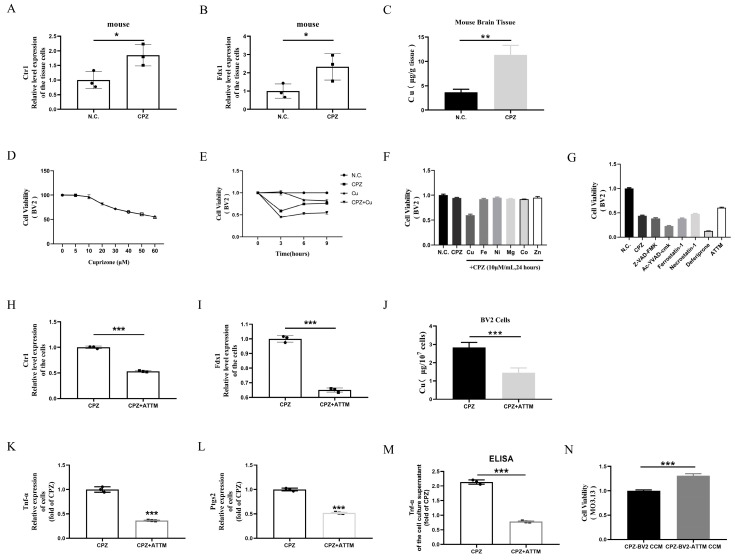
Cuprizone forms a metal copper ketone complex with copper, causing cellular copper intoxication and promoting the formation of pro-inflammatory microglia. (**A**,**B**) Relative gene expression changes of *Ctr1* and *Fdx1* in brain tissue of Cuprizone-treated mice. (**C**) Copper content in brain tissue of Cuprizone-treated mice. (**D**) Viability trends of BV2 cells stimulated with Cuprizone at concentrations of 0, 5, 10, 20, 30, 40, 50, 60 μM for 24 h. (**E**) Viability trends of BV2 cells stimulated with 20 μM Cuprizone, copper, and Cuprizone–copper for 0, 3, 6, 9 h. (**F**) Viability trends of BV2 cells treated with different metal ions at a concentration of 10 μM along with 10 μM/mL Cuprizone for 24 h. (**G**). Viability trends of BV2 cells treated with different cell death inhibitors after 24 h of treatment with 20 μM Cuprizone-copper. (**H**,**I**) Relative gene expression changes of *Ctr1* and *Fdx1* in BV2 cells treated with ATTM after Cuprizone exposure. (**J**) Copper content in BV2 cells treated with ATTM after Cuprizone exposure. (**K**–**M**) ATTM treatment inhibits the formation of pro-inflammatory phenotypes in microglia. (**N**) Effects of conditioned medium from ATTM-treated microglia on cell viability in MO3.13 cells. * *p* < 0.05, ** *p* < 0.01, *** *p* < 0.001. All experiments were independently repeated three times.

To assess the expression of copper content in the brain tissues of Cuprizone-fed mice, we first observed significantly elevated gene expression levels of *Ctr1* (A high-affinity Cu transporter) and *Fdx1* (A copper death marker) in the brain tissues of Cuprizone-fed mice (Figure 4A,B). Additionally, copper content analysis revealed significantly increased copper levels in the brain tissues of Cuprizone-fed mice (Figure 4C). Next, we conducted in vitro validations using a concentration gradient of Cuprizone (0–60 μM/mL) to stimulate BV2 cells for 24 h. Results showed a negative correlation between Cuprizone concentration and cell viability (Figure 4D). Although Cuprizone caused a decrease in cell viability, this effect was not pronounced, even with increasing concentrations.

Based on Chinese and international drinking water quality standards for copper content (1.0 mg/L and 2 mg/L, equivalent to 15.625 μmol/L and 31.250 μmol/L, respectively), we selected 20 μmol/L as the experimental concentration. Results showed that the cytotoxic effects of the copper(II) bis(cyclohexanone) oximate complex were more significant than those of Cuprizone or copper alone (Figure 4E). Furthermore, we treated BV2 cells with Cuprizone in combination with various metal ion salts for 24 h. Significant cytotoxic effects were only observed when Cuprizone was combined with CuCl_2_, not with other metal ions (Figure 4F). To investigate cell type specificity, we applied the same treatment to MO3.13. Consistent with previous results, significant cytotoxic effects were only observed in cells treated with Cuprizone combined with CuCl_2_ (Figure S2A–C).

These findings indicate that Cuprizone’s cytotoxic effects are specifically associated with the formation of copper(II) bis(cyclohexanone) oximate complexes. To determine if copper-induced cell death mechanisms are involved, we treated Cuprizone-copper complex-stimulated MO3.13 and BV2 cells with various cell death inhibitors: Z-VAD-FMK (apoptosis inhibitor), Ac-YVAD-cmk (pyroptosis inhibitor), Ferrostain-1 (ferroptosis inhibitor/lipid peroxidase inhibitor), Necrostain-1 (necroptosis inhibitor), Deferiprone (ferroptosis inhibitor/iron chelator), and ATTM (cuproptosis inhibitor/copper chelator). Results showed that only ATTM pretreatment significantly rescued cell viability in all cell lines, while other inhibitors exhibited varying effects across different cell types (Figure 4G and Figure S2D–F). Additionally, ATTM treatment partially reversed the copper(II) bis(cyclohexanone) oximate complex-induced elevation of *Ctr1* and *Fdx1* expression (Figure 4H,I). Importantly, cellular copper content analysis showed that the copper(II) bis(cyclohexanone) oximate complex increased intracellular copper levels (Figure 4J).

Finally, ATTM treatment of BV2 cells significantly inhibited the gene expression of pro-inflammatory cytokines TNF-α and Ptgs2 (Figure 4K,L). ELISA analysis confirmed that ATTM effectively reduced TNF-α secretion into the culture medium by BV2 cells (Figure 4M). We co-cultured MO3.13 oligodendrocytes with conditioned medium collected from ATTM-treated BV2 cells for 24 h. Increased cell viability in MO3.13 cells (Figure 4N).

### 3.4. Scutellarin Treatment Restores Mitochondrial Dysfunction in BV2 Cells Induced by Cuprizone–Copper(II) Complexes

**Figure 5:**
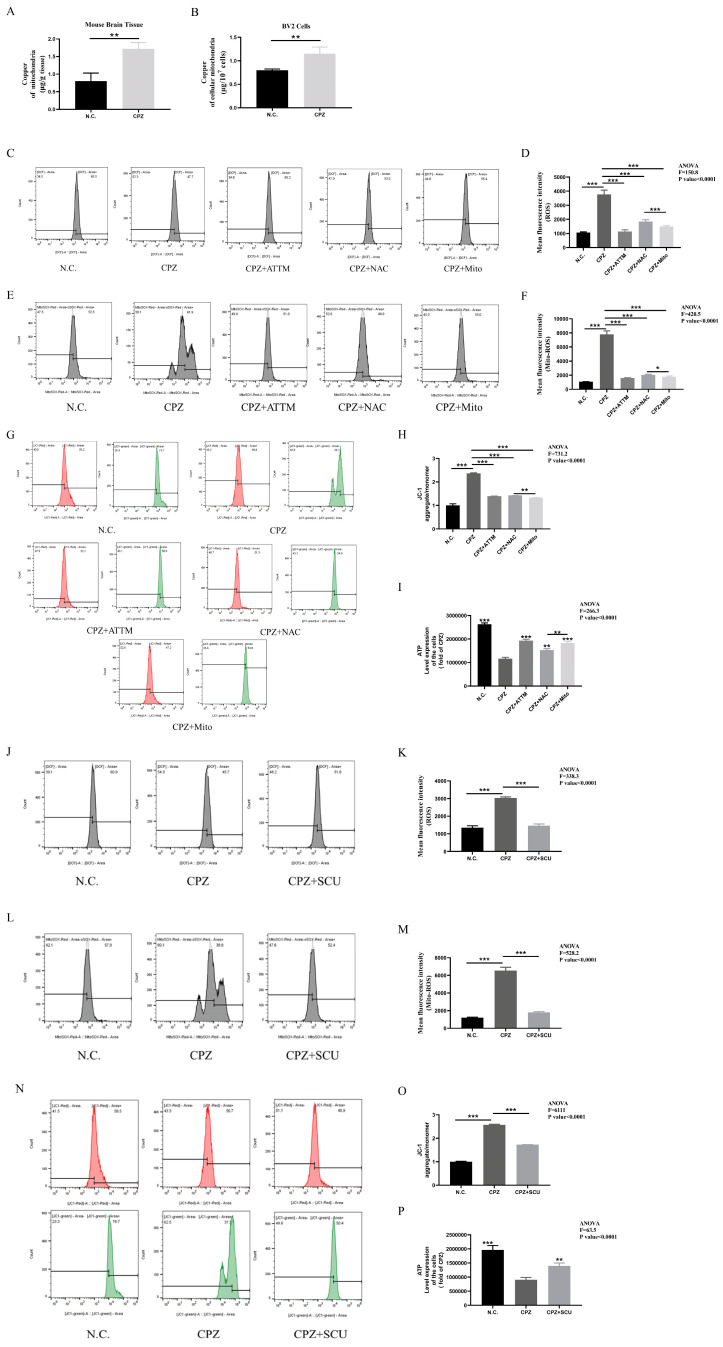
Scutellarin treatment restores mitochondrial dysfunction in BV2 cells caused by Cuprizone. (**A**,**B**) Copper content in mitochondria of brain tissue from Cuprizone-fed mice and in BV2 cells treated with Cuprizone. (**C**–**I**) Changes in mitochondrial reactive oxygen species, cellular reactive oxygen species, mitochondrial membrane potential, and ATP in BV2 cells treated with ATTM, NAC, and Mito after Cuprizone exposure. (**J**–**P**) Changes in mitochondrial reactive oxygen species, cellular reactive oxygen species, mitochondrial membrane potential, and ATP in BV2 cells treated with Scutellarin after Cuprizone–copper exposure. * *p* < 0.05, ** *p* < 0.01, *** *p* < 0.001. All experiments were independently repeated three times.

Next, copper content analysis of mitochondria isolated from brain tissues and BV2 cells revealed significantly increased copper levels (Figure 5A,B). Transmission electron microscopy showed mitochondrial shrinkage in Cuprizone–copper complex-treated BV2 cells, which was alleviated by ATTM treatment (Figure S3).

Having observed morphological changes, we next investigated functional alterations by measuring cellular reactive oxygen species (ROS), mitochondrial ROS, and mitochondrial membrane potential. Results showed that Cuprizone treatment increased cellular and mitochondrial ROS levels and decreased mitochondrial membrane potential. These effects were reversed by ATTM, NAC (cellular ROS scavenger), and Mito-TEMPO (mitochondrial ROS scavenger) treatment (Figure 5C–H). Notably, Mito-TEMPO was more effective than NAC in reducing both mitochondrial and cellular ROS levels, suggesting that mitochondrial ROS elevation precedes cellular ROS increase after Cuprizone treatment (Figure 5F–K). We further measured cellular ATP levels and found that Cuprizone treatment significantly reduced ATP content, which was restored by ATTM, NAC, and Mito-TEMPO treatment. Consistent with previous results, Mito-TEMPO was more effective than NAC in restoring ATP levels (Figure 5I). These findings confirm that Cuprizone–copper complex treatment disrupts mitochondrial function and that mitochondrial damage precedes ROS elevation, rather than vice versa.

Based on these findings, we investigated the effects of scutellarin on mitochondrial function. Results showed that scutellarin treatment inhibited Cuprizone-induced ROS elevation, restored mitochondrial membrane potential, and increased ATP levels in BV2 cells (Figure 5J–P). These findings demonstrate that scutellarin restores mitochondrial function in BV2 cells.

### 3.5. Cuprizone–Copper(II) Complexes Promote Pro-Inflammatory Microglial Activation by Increasing Mitochondrial ROS

**Figure 6:**
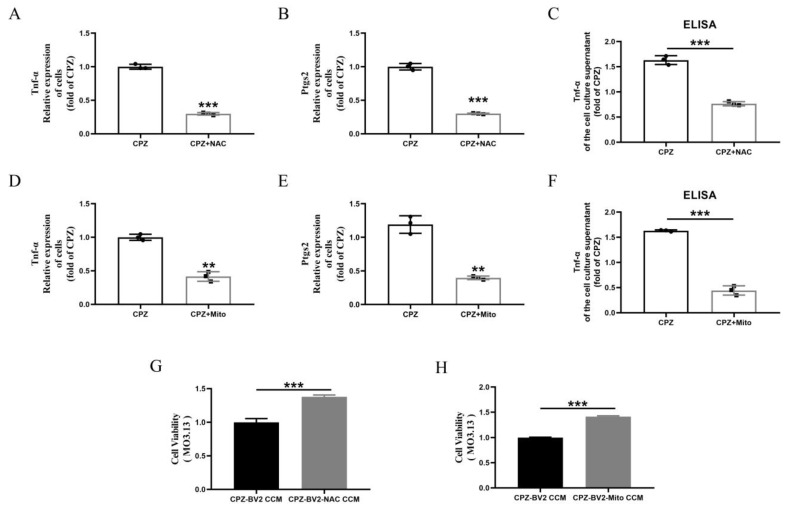
In vitro, NAC and Mito can inhibit Cuprizone-induced pro-inflammatory microglia activation and alleviate myelin cell damage. (**A**–**F**) NAC and Mito treatment inhibits the formation of pro-inflammatory phenotypes in microglia. (**G**,**H**) Effects of conditioned medium from NAC-treated and Mito-treated microglia on cell viability in MO3.13 cells. ** *p* < 0.01, *** *p* < 0.001. All experiments were independently repeated three times.

Finally, we examined the effects of NAC and Mito-TEMPO on pro-inflammatory cell formation in Cuprizone–copper complex-treated cells. Both treatments reduced TNF-α and Ptgs2 gene expression and TNF-α secretion into the culture medium (Figure 6A–F). Similarly, these treatments increased viability in MO3.13 cells (Figure 6G,H). These results indicate that NAC and Mito-TEMPO inhibit pro-inflammatory microglial activation. They also provide reverse evidence that Cuprizone–copper complexes induce pro-inflammatory phenotypes via mitochondrial ROS elevation, ultimately leading to myelin cell damage.

### 3.6. Scutellarin Inhibits Pro-Inflammatory Microglial Responses by Reducing Cuprizone–Copper Complex-Induced Mitochondrial Damage and Lipid Peroxidation

**Figure 7:**
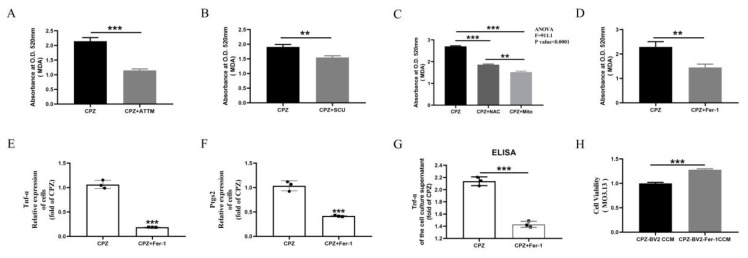
In vitro, inhibition of pro-inflammatory responses in microglia by reducing Cuprizone-induced lipid oxidation. (**A**–**D**) ATTM, Scutellarin, NAC, Mito, and Fer-1 treatment inhibits the increase in MDA in Cuprizone-induced microglia. (**E**–**G**) Fer-1 treatment inhibits the formation of pro-inflammatory phenotypes in microglia. (**H**) Effects of conditioned medium from Fer-1-treated microglia on cell viability in MO3.13 cells. ** *p* < 0.01, *** *p* < 0.001. All experiments were independently repeated three times.

Lipid peroxidation plays a critical role in Cuprizone-induced demyelination [28,31,33]. We observed that the Cuprizone–copper complex promoted MDA accumulation in BV2 cells, which was reduced by ATTM treatment (Figure 7A). Similarly, scutellarin treatment inhibited MDA accumulation (Figure 7B). Since lipid peroxidation depends on ROS elevation, which originates from mitochondria, we treated cells with NAC and Mito-TEMPO. Both treatments inhibited MDA accumulation, with Mito-TEMPO being more effective than NAC (Figure 7C).

Ferrostain-1, a lipid peroxidation inhibitor, reduced MDA levels in Cuprizone–copper complex-stimulated BV2 cells (Figure 7D). Ferrostain-1 treatment also reduced TNF-α and Ptgs2 gene expression and TNF-α secretion into the culture medium (Figure 7E–G). Similarly, conditioned medium from Ferrostain-1-treated cells alleviated the reduced viability in MO3.13 cells (Figure 7H). These findings indicate that Ferrostain-1 inhibits pro-inflammatory microglial activation and provide reverse evidence that Cuprizone–copper complexes induce pro-inflammatory phenotypes via lipid peroxidation.

### 3.7. Scutellarin Inhibits Cuprizone–Copper Complex-Induced Pro-Inflammatory Microglial Activation via the p38MAPK/TNF-α Signaling Pathway

**Figure 8:**
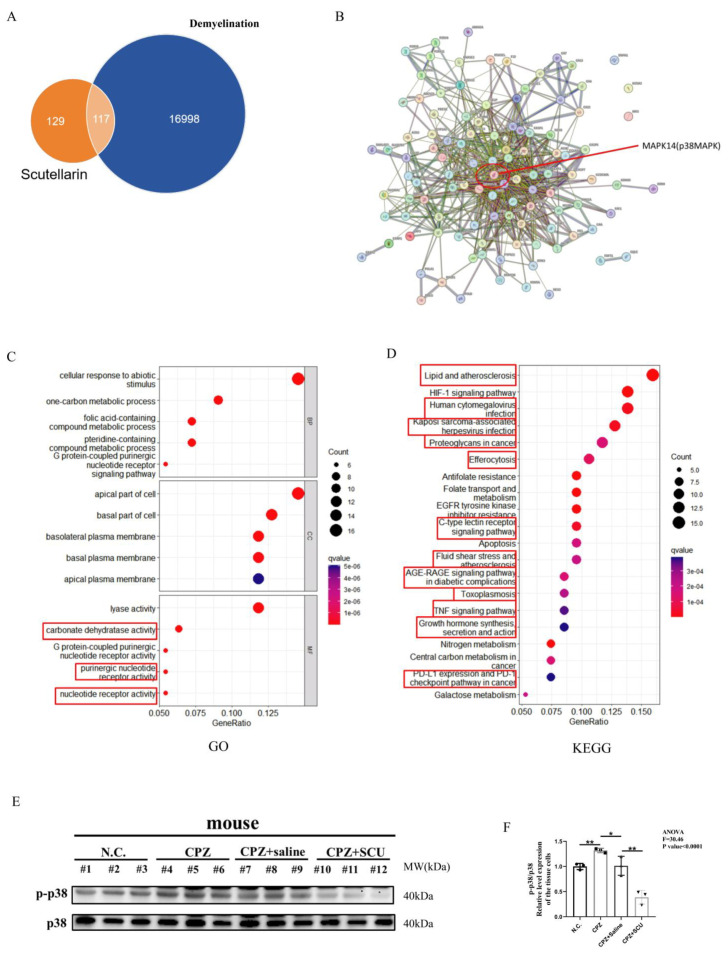
Screening for overlapping molecular mechanisms seen in demyelination and responsive to scutellarin. (**A**) Venn diagram of predicted targets of Scutellarin and predicted targets of demyelination. (**B**) STRING predicts protein–protein interaction. (**C**) GO enrichment analysis of predicted targets of Scutellarin treatment for demyelination. Red boxes revealed significant enrichment in mitochondrial functions. (**D**) KEGG pathway analysis of predicted targets of Scutellarin treatment for demyelination. Red boxes showed that these pathways were related to p38MAPK. (**E**,**F**) Western blotting analysis of p38 protein phosphorylation levels in brain tissue of mice after Scutellarin treatment. * *p* < 0.05, ** *p* < 0.01. Three mouse per group.

To further investigate the molecular targets of scutellarin in demyelination therapy, we screened potential targets of scutellarin and multiple sclerosis using the SwissTargetPrediction (http://www.swisstargetprediction.ch/, 6 December 2024) and GeneCards databases (https://www.genecards.org/, 6 December 2024). p38MAPK was identified as a candidate target, while ERK and JNK were not. Based on previous experimental results and literature reviews [62], we selected p38MAPK for further study due to its critical role in multiple sclerosis. We uploaded the identified targets to the Metascape database for GO and KEGG analysis. GO analysis revealed significant enrichment in mitochondrial functions, consistent with our previous findings. KEGG analysis showed that 2/3 of the top 20 pathways were related to p38MAPK, further highlighting its importance in scutellarin’s therapeutic effects on multiple sclerosis (Figure 8A–D).

To validate these predictions, Western blotting analysis showed that while total p38MAPK protein levels remained unchanged, phosphorylated p38MAPK (p-p38MAPK) levels were significantly elevated in the brain tissues of Cuprizone-fed mice compared to controls. Scutellarin treatment inhibited this elevation in p-p38MAPK levels (Figure 8E,F). p-p38MAPK fluorescence intensity was increased by Cuprizone feeding and reduced by scutellarin treatment. Co-localization of p-p38MAPK and Iba1 fluorescence indicated that Cuprizone induces microglial activation via p38MAPK phosphorylation (Figure S4A–C).

### 3.8. Reduced p38MAPK Phosphorylation Inhibits Pro-Inflammatory Cell Formation

**Figure 9:**
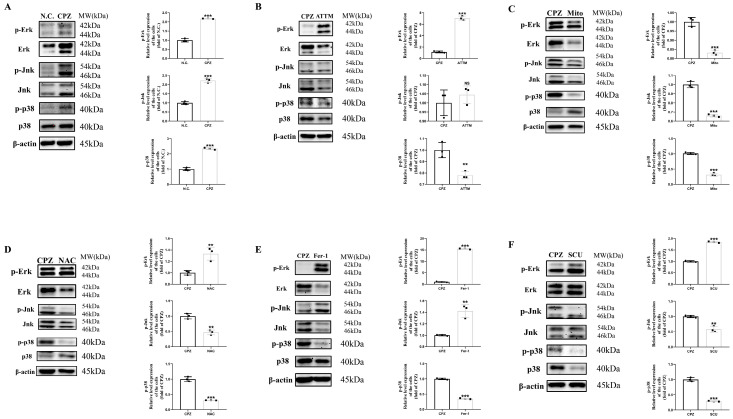
Changes in protein expression of MAPKs in BV2 cells treated with Cuprizone. (**A**–**F**). Western blotting analysis of changes in protein expression levels of MAPKs in BV2 cells treated with ATTM, Scutellarin, NAC, Mito, and Fer-1 after Cuprizone exposure. ^NS^ *p* > 0.05, ** *p* < 0.01, *** *p* < 0.001. All experiments were independently repeated three times.

After treating Cuprizone–copper complex-stimulated BV2 cells with ATTM, Mito-TEMPO, NAC, Fer-1, and SCU, only p38MAPK phosphorylation was consistently and effectively inhibited across three independent experiments, while ERK and JNK expression patterns were inconsistent and unstable (Figure 9A–F). These findings further support the critical role of p38MAPK phosphorylation in demyelinating related diseases.

**Figure 10:**
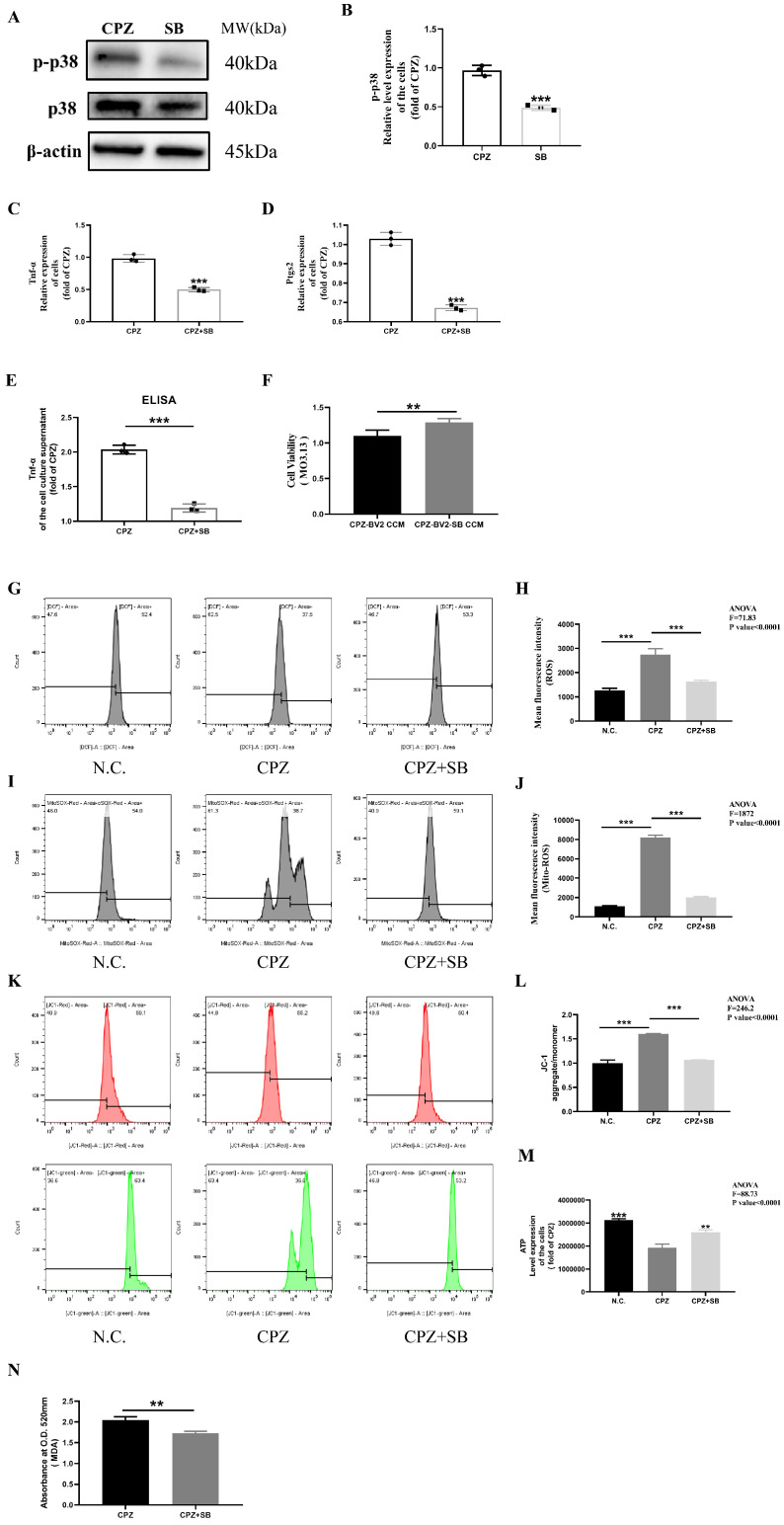
In vitro, reduction in p38 MAPK phosphorylation levels inhibits the formation of Cuprizone-induced pro-inflammatory cells. (**A**,**B**) Western blotting analysis of p38 MAPK protein phosphorylation in BV2 cells treated with SB203580 after Cuprizone exposure. (**C**–**E**) SB203580 treatment inhibits the formation of pro-inflammatory phenotypes in microglia. (**F**) Effects of conditioned medium from SB203580-treated microglia on cell viability in MO3.13 cells. (**G**–**N**) Changes in mitochondrial reactive oxygen species, cellular reactive oxygen species, mitochondrial membrane potential, ATP and MDA in BV2 cells treated with SB203580 after Cuprizone exposure. ** *p* < 0.01, *** *p* < 0.001. All experiments were independently repeated three times.

Next, we treated Cuprizone–copper complex-stimulated BV2 cells with SB203580, a p38MAPK inhibitor. SB203580 treatment reduced both total and phosphorylated p38MAPK protein levels, confirming successful inhibition of p38MAPK activity (Figure 10A,B). SB203580 treatment also reduced TNF-α and Ptgs2 gene expression and TNF-α secretion by BV2 cells (Figure 10C–E). Similarly, SB203580 treatment alleviated the reduced viability in MO3.13 cells (Figure 10F). Finally, mitochondrial function analysis showed that SB203580 inhibited mitochondrial dysfunction in BV2 cells (Figure 10G–N).

Collectively, these findings demonstrate that scutellarin inhibits Cuprizone-induced demyelination by reducing p38MAPK phosphorylation, restoring mitochondrial function in microglia, and suppressing the pro-inflammatory activity.

## 4. Discussion

Demyelination of the central nervous system (CNS) leads to neurodegenerative diseases that can jeopardize human health and affect the development of society. There are various mechanisms for demyelination, one of which is the damage to oligodendrocytes caused by inflammatory mediators produced by microglia [50,51,52,53].

The CPZ mouse model is a classic toxic demyelination model widely used to study demyelination in the central nervous system [12,13]. Although studies have shown that CPZ can cause demyelination in mice, research on the potential cellular and molecular mechanisms of CPZ remains scarce and deserves further investigation. CPZ is a copper ion chelating agent that can form dark blue metal copper ketone complexes with copper (II) [21]. Since the copper ketone reaction of CPZ can be used to identify the presence of copper in colorimetric detection, it is often used in public health and environmental testing to measure whether the copper content of substances exceeds the standard. Studies have shown that the demyelinating effect of CPZ is not due to copper chelation, but rather to acute excessive copper poisoning caused by the formation of metal copper ketone complexes with copper [22]. However, that study only used treatments with other copper chelating agents, focusing only on the phenotype, and the more detailed mechanisms have not been thoroughly investigated.

Here, we discovered and elucidated a new pathological and molecular mechanism in which metal copper ketone complexes formed by CPZ and copper act as a medium to cause metal poisoning in the central nervous system, which in turn activates the occurrence and development of neuroinflammation, leading to myelin damage and loss.

Here, our study demonstrated that CPZ can induce copper homeostasis imbalance in microglia, causing immune damage to myelin cells. In in vivo experiments, we found that the copper content level in the brain tissue of the CPZ-induced toxic demyelination mouse model was imbalanced and in particular, the copper homeostasis in mitochondria was the most severely imbalanced. In in vitro experiments, we found that CPZ can form metal copper ketone complexes with copper, causing acute metal copper poisoning in cells, which activates cell copper death and induces damage to neuroglial cells. The synergistic effect of the copper ketone reaction between CPZ and copper can induce a decrease in cell viability of neuroglial cells and exacerbate cell damage. Compared with other metal elements, the cell damage induced by CPZ treatment is only strongly correlated with copper.

Exposure to CPZ–copper ketone complexes induced the activation of pro-inflammatory phenotypes in microglia, which can be reflected by the transcriptional level measurement of inflammatory-related biomarkers. In this study, we used a conditioned medium co-culture method to study the killing ability of microglial secretions on myelin cells. Under the premise of excluding the direct effect of metal copper ketone complexes formed by CPZ on myelin cells, we found that the cell supernatant of microglia collected after exposure to CPZ–copper ketone complexes can damage the cell viability of oligodendrocytes. ATTM, a specific copper ion chelating agent and the first-line clinical medication for Wilson’s disease (WD, a genetic disease with abnormal deposition of copper ions in the body), can effectively rescue this phenomenon. Other studies have shown that ATTM has a good therapeutic effect in another mouse model of myelin loss, experimental allergic encephalomyelitis (EAE), which is a demyelinating disease model [65]. Therefore, copper is a poor prognostic marker for demyelinating diseases, which is complementary to our observations in environmental, clinical, and animal models.

Based on the above, we also propose new questions: Can copper homeostasis imbalance first cause damage to myelin cells, and then trigger an inflammatory response in microglia, thereby promoting the development of demyelination? In addition, copper is an essential trace element for organisms, widely distributed in biological tissues, mostly in the form of organic complexes, so the biological internal environment is rich in copper. Cuprizone is ingested by mice through feeding, then transported to the blood through the digestive system of the mice, and then transported to the brain through the cardiopulmonary system. During this process, it must have combined with copper, and the resulting effect is definitely not the same as that of Cuprizone alone. Therefore, detecting the presence, stability, and distribution of metal copper ketone complexes in mice will be the goal of our further in-depth investigation in the future.

Secondly, further in vitro studies have shown that CPZ–copper complexes activate inflammatory injury by inducing mitochondrial damage causing ROS generation. In vitro, CPZ–copper complexes promote elevated levels of mitochondrial copper in BV2 cells, causing mitochondrial crumpling, which results in the dysfunction of the BV2 cell’s mitochondria, leading to the generation of large amounts of ROS to activate and exacerbate the BV2 cell’s inflammatory response. In addition, the use of ATTM, the oxidative stress inhibitor NAC and the mitochondrial reactive oxygen species inhibitor Mito can be very effective in restoring mitochondrial dysfunction and reducing the level of ROS in cells.

Third, CPZ–copper complexes activate inflammatory responses by promoting lipid oxidation in microglia in vitro. Abnormalities in lipid oxidation have been reported to be involved in the occurrence and development of demyelination. Previous studies have shown that microglia cause abnormalities in lipid oxidation through ferroptosis, thereby aggravating the progression of demyelination [66]. In this study, we determined in a BV2 cell model that CPZ–copper complexes can directly mediate the transformation of pro-inflammatory cells. In the study, we found that with the increase in the intake of CPZ–copper complexes by microglia in vitro, the level of lipid oxidation in cells can be increased to a certain extent, thereby promoting the formation of pro-inflammatory cell phenotypes. Further rescue experiments showed that treatment with the copper chelating agent ATTM and the lipid oxidation inhibitor Fer-1 can significantly inhibit the induction and promotion of inflammation by CPZ–copper complexes, reduce intracellular MDA levels, and decrease immune responses, thereby confirming the relevance of this finding. Treatment with NAC and Mito can also reduce intracellular MDA levels and decrease the formation of pro-inflammatory cells. Since the lipid oxidation inhibitor Fer-1 cannot inhibit the formation of mitochondrial reactive oxygen species [67] and CPZ–copper ketone complexes can induce mitochondrial damage, it cannot be proven that lipid oxidation is caused by the increase in mitochondrial reactive oxygen species due to mitochondrial damage. However, studies on the treatment with NAC and Mito can prove this point.

Fourth, Scutellarin is a flavonoid drug widely used in the treatment of neurological diseases. Scutellarin is widely distributed in the brain after entering it, thereby exerting protective effects. Scutellarin can reduce vascular resistance, increase the permeability of the blood–brain barrier (BBB), and improve the microenvironment of brain tissue [55]. The protective effects of Scutellarin on cerebral infarction and Alzheimer’s disease have been studied. Scutellarin can cross the blood–brain barrier, reduce the release of nitric oxide in brain tissue, and reduce the infarct area in the brain [68]. In addition, Scutellarin can inhibit the production and deposition of β-amyloid protein (Aβ), protect neurons from oxidative stress damage, and thereby alleviate neurodysfunction in Alzheimer’s disease [60]. However, the therapeutic effects and underlying mechanisms of Scutellarin in demyelinating related diseases are still unclear. However, there is no clear report on how this drug treats neuroinflammation induced by abnormal lipid oxidation in microglia. In in vivo experiments, we found that after treatment with Scutellarin, the inflammatory demyelination phenomenon in the mouse brain was alleviated. In in vitro experiments, the mitochondrial dysfunction of microglia induced by CPZ–copper complexes was restored, lipid oxidation was reduced, and the expression of inflammatory mediators was decreased. This is crucial for our treatment and repair of nerve damage in demyelinating related diseases. More importantly, this study may provide a new therapeutic direction for neuroinflammation mediated by microglia using lipid oxidation as a target. In summary, these results indicate that Scutellarin may be an effective candidate drug for the treatment of demyelinating diseases such as multiple sclerosis. But how can the in vitro results be extrapolated to the in vivo system? What is the pro-inflammatory state and possible protective effects of baicalein in vivo? What is the mitochondrial function and ROS status in vivo after baicalein treatment? Next, we need further in vivo experiments to verify the above ideas.

Fifth, the formation of pro-inflammatory cell phenotypes by CPZ–copper complexes is achieved by targeting the MAPKs signaling pathway. Based on previous experimental results and a literature review, we selected the MAPK signaling pathway and conducted mechanistic verification. In our study, the results showed that stimulation with CPZ–copper complexes can activate the phosphorylation of the MAPK signaling pathway in BV2 cells in vitro. However, under the treatment of ATTM, Fer-1, NAC, Mito, and SCU, only the total protein level and phosphorylation level of p38 were stably and effectively inhibited. Therefore, we chose p38 as the main research direction. In vitro experiments, the p38 inhibitor SB203580 can inhibit the increase in phosphorylation levels of p38 induced by CPZ–copper complexes, reduce lipid oxidation, prevent the transition of cell phenotype to inflammatory phenotype, and thereby alleviate damage to myelin cells. Overall, we show that the immune response mediated by BV2 cells to CPZ–copper complexes is achieved by targeting the p38MAPK signaling pathway in vitro.

In fact, the Cuprizone-induced demyelination model in mice is not a traditional MS animal model. It is well known that there are three types of animal modeling for studying MS: 1. Cuprizone-induced mouse demyelination model; 2. EAE mouse demyelination model; and 3. use of, e.g., LPS-induced mouse demyelination model. Therefore, what is the therapeutic efficacy of scutellarin on other traditional mouse demyelination models? Although scutellarin treatment of LPS-induced demyelination in animals has not been reported, studies on scutellarin treatment of EAE models have been reported. Yuan et al. reported that scutellarin alleviates experimental autoimmune encephalomyelitis by suppressing pathogenetic CXCR6+ CD4 cells [63]. Because fewer studies have been reported, further investigation is needed regarding the therapeutic efficacy of scutellarin in different animal models of demyelination. To sum up, the Cuprizone-induced mouse demyelination model is only one of the ways to study demyelination, and it is not a substitute for MS or even other demyelination-related diseases.

In conclusion, studying the progression of demyelination using the demyelination model established by Cuprizone and elucidating the therapeutic effects and mechanisms of Scutellarin carriers in myelin damage may bring new measures for the treatment of demyelinating related diseases, which are not only innovative but also have important implications for clinical translation and provide great reference for the application of other antioxidant drugs in demyelinating related diseases.

## 5. Conclusions

In summary, in vivo studies have shown the neuroprotective effects of Scutellarin on CPZ mice and that the mechanism is closely related to the improvement of mitochondrial dysfunction and the reduction in lipid oxidation damage by inhibiting the p38MAPK pathway in microglia. These findings provide solid data support for Scutellarin as a candidate drug for the clinical treatment of demyelinating-related diseases.

## Data Availability

The data presented in this study are available on request from the corresponding author. The data are not publicly available due to confidentiality issues.

## Supplementary Materials

The following supporting information can be downloaded at: https://www.mdpi.com/article/10.3390/antiox14060723/s1, Figure S1: (A). Immunofluorescence observation of myelin in the brain of Cuprizone-treated mice (hippocampus and corpus callosum, zoom 5X, scale bars = 200 μm). (B). Immunofluorescence observation of microglia in the brain of Cuprizone-treated mice. (hippocampus and corpus callosum, zoom 36X, scale bars = 50 μm). (C). Immunofluorescence observation of myelin damage in the brain of Scutellarin-treated mice (hippocampus and corpus callosum, zoom 5X, scale bars = 200 μm). ** *p* < 0.01, *** *p* < 0.001. 3 mouse per group. Figure S2: (A). Viability trends of MO3.13 cells treated with different metal ions at a concentration of 10 μM along with 10 μM Cuprizone for 24 h. (B). Viability trends of MO3.13 cells treated with various cell death inhibitors after 24 h of treatment with 20 μM Cuprizone-copper. Figure S3: Transmission electron microscopy observation of mitochondrial morphological changes in BV2 cells treated with Cuprizone. The arrows point to the mitochondria. (scale bars are 1 μm and 50 nm respectively). Figure S4: (A–C). Immunofluorescence observation of p-p38 fluorescence expression levels in brain tissue of mice after Scutellarin treatment. (zoom 36X, scale bars = 50 μm). * *p* < 0.05, ** *p* < 0.01. 3 mouse per group. Table S1: Sequences of primers. Table S2: Results of ANOVA tests.
