# Supplementary material for: Scutellarin Alleviates Cuprizone-Induced Demyelination by Improving Mitochondrial Dysfunction, Reducing Lipid Oxidation and Inhibiting the p38 MAPK Pathway
Source: Antioxidants (Basel). 2025 Jun 12;14(6):723. doi: 10.3390/antiox14060723 (PMC12189410; doi:10.3390/antiox14060723)
Supplement: Supplementary file 1 [file antioxidants-14-00723-s001.zip › Supplementary Figure.pdf]

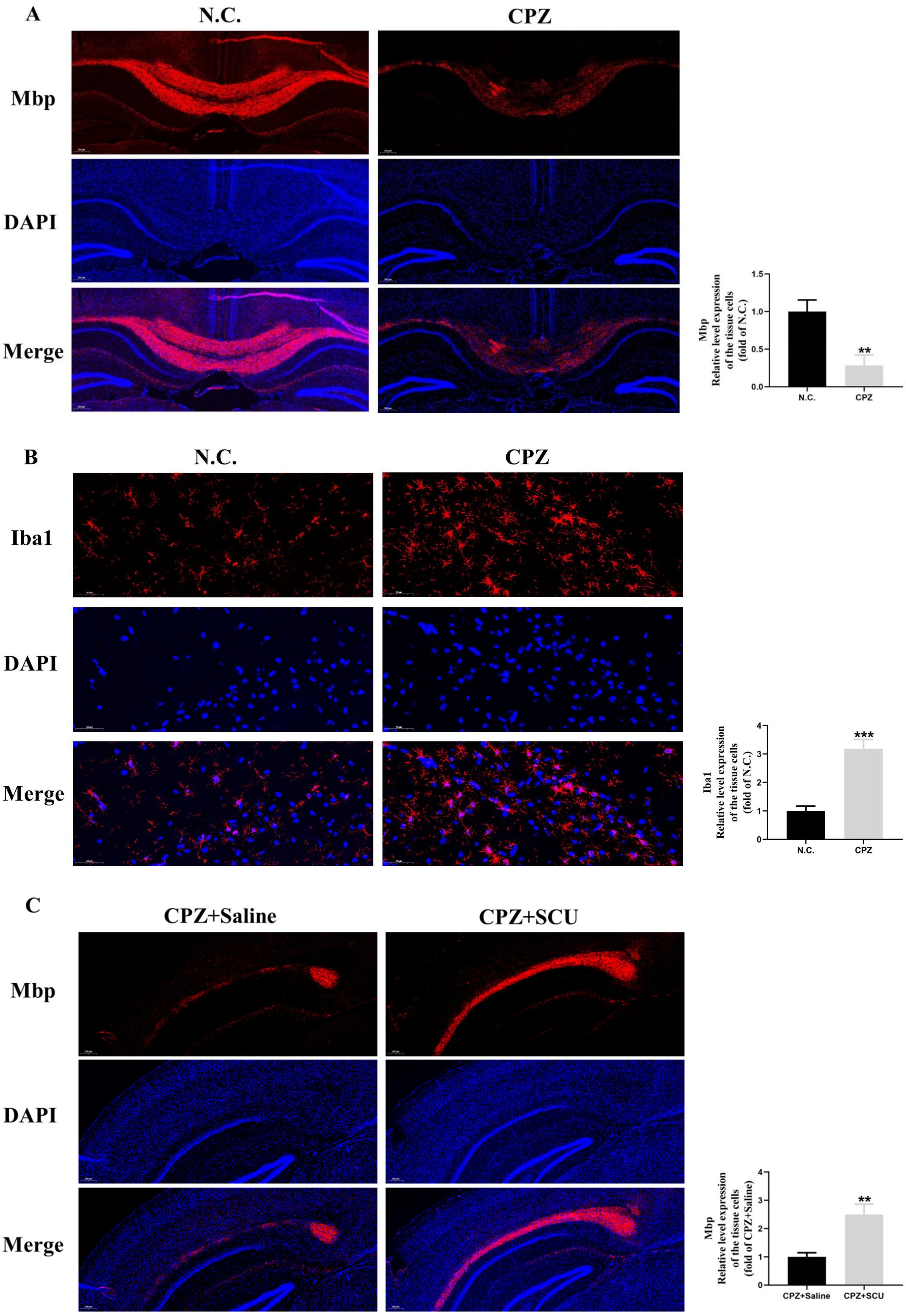
A
N.C.
CPZ
Mbp
DAPI
Merge
Mbp
Relative level expression
of the tissue cells
(fold of N.C.)
1.5
1.0
0.5
0.0
**
N.C.
CPZ
B
N.C.
CPZ
Iba1
DAPI
Merge
Iba1
Relative level expression
of the tissue cells
(fold of N.C.)
4
3
2
1
0
***
N.C.
CPZ
C
CPZ+Saline
CPZ+SCU
Mbp
DAPI
Merge
Mbp
Relative level expression
of the tissue cells
(fold of CPZ+Saline)
4
3
2
1
0
**
CPZ+Saline
CPZ+SCU

Figure S1. (A). Immunofluorescence observation of myelin in the brain of Cuprizone-treated mice (hippocampus and corpus callosum, zoom 5X, scale bars=200μm). (B). Immunofluorescence observation of microglia in the brain of Cuprizone-treated mice. (hippocampus and corpus callosum, zoom 36X, scale bars=50μm). (C). Immunofluorescence observation of myelin damage in the brain of Scutellarin-treated mice (hippocampus and corpus callosum, zoom 5X, scale bars=200μm).. ** p <0.01, *** p <0.001. 3 mouse per group.

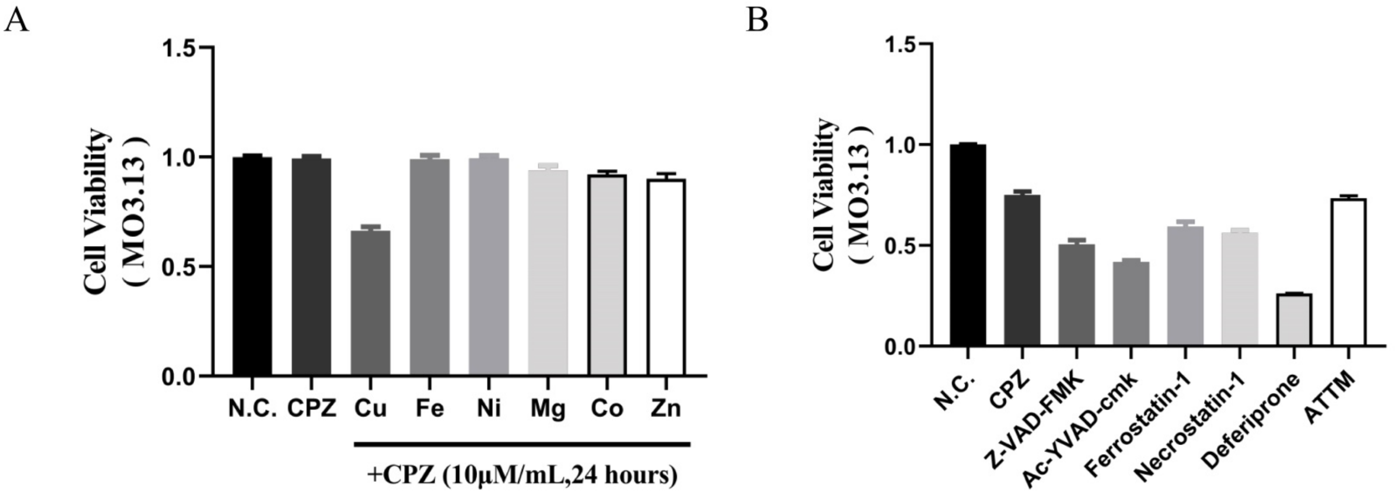

Figure S2. (A). Viability trends of MO3.13 cells treated with different metal ions at a concentration of 10 μM along with 10 μM Cuprizone for 24 hours. (B). Viability trends of MO3.13 cells treated with various cell death inhibitors after 24 hours of treatment with 20 μM Cuprizone-copper.

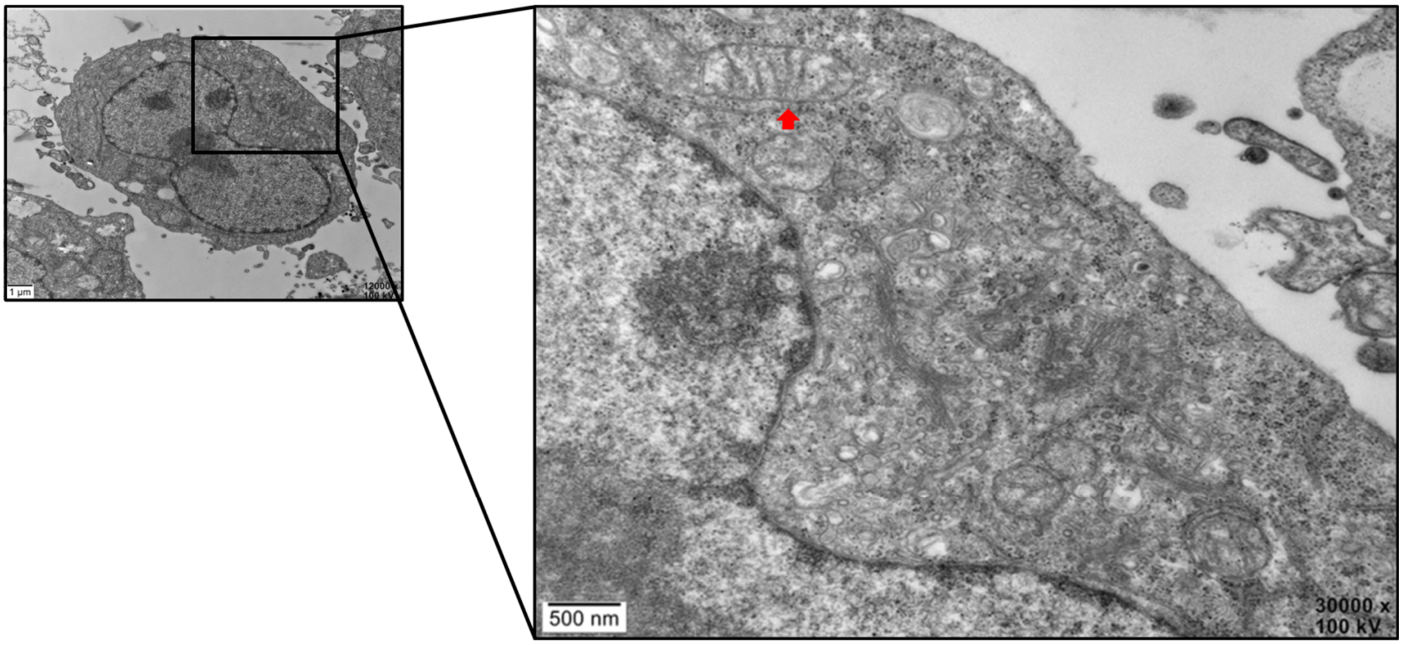

N.C.

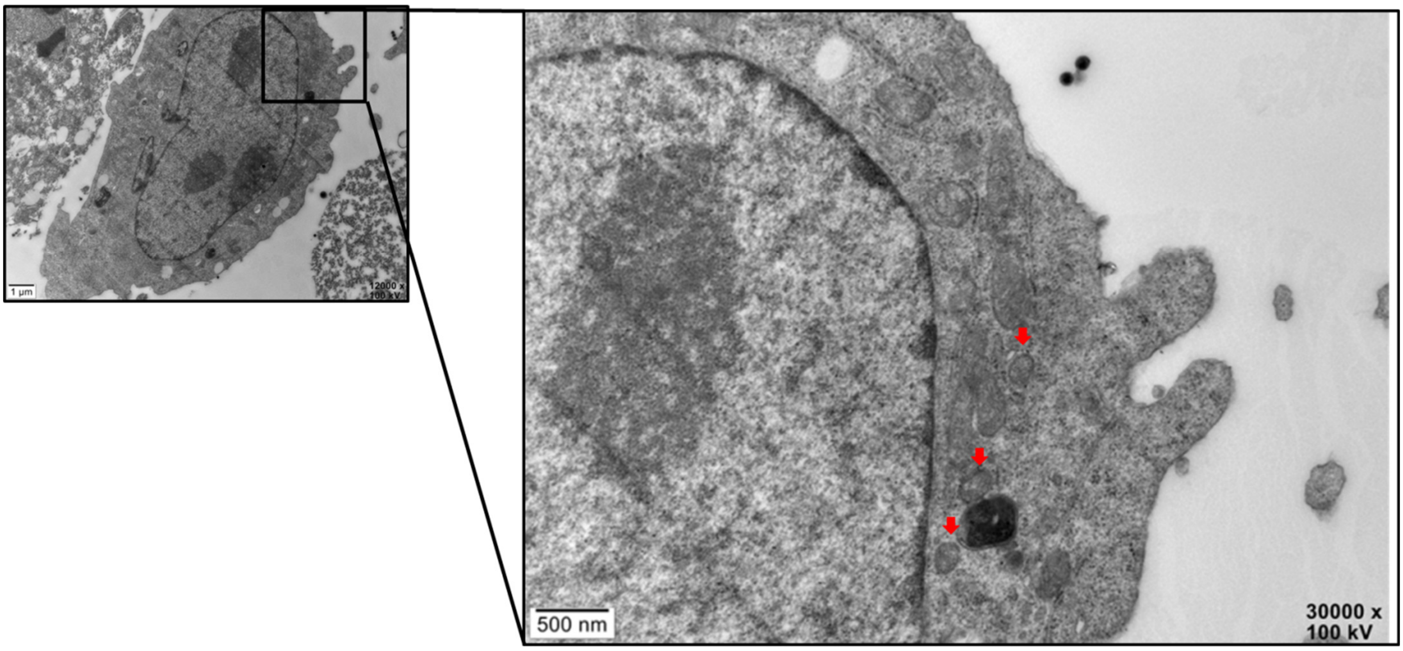

CPZ

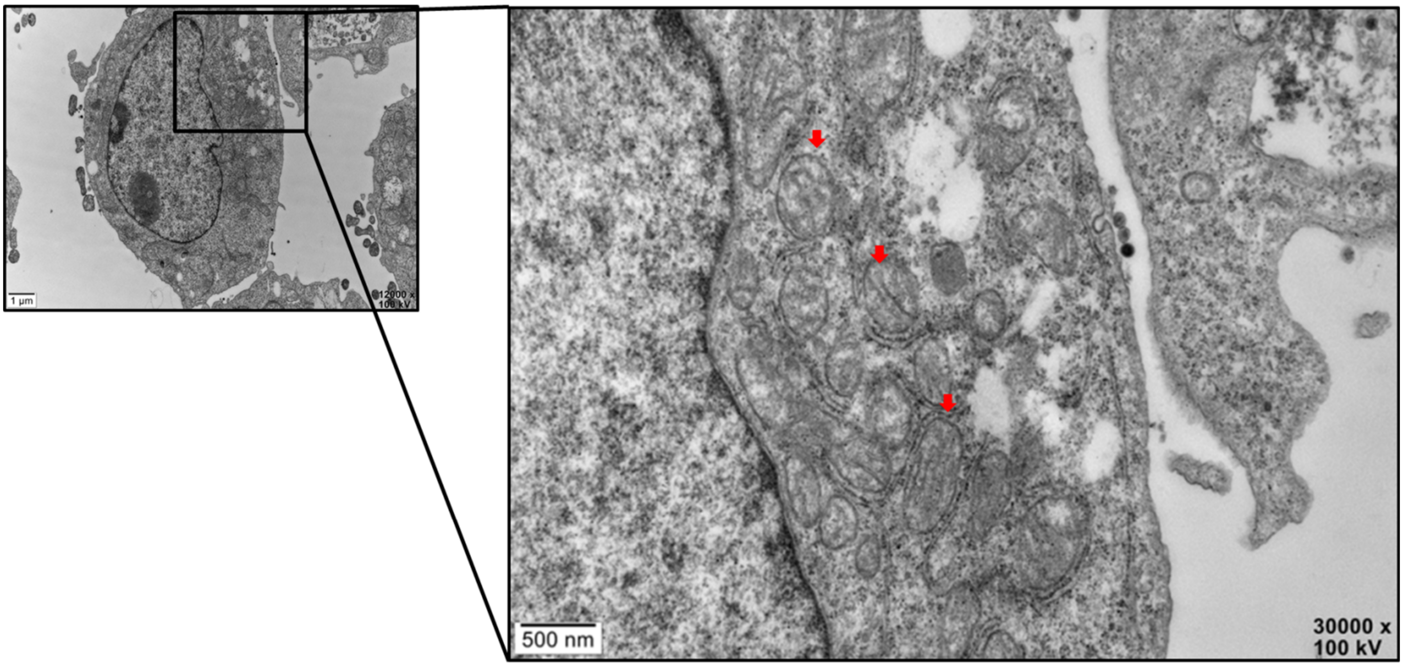

CPZ+ATTM

Figure S3. Transmission electron microscopy observation of mitochondrial morphological changes in BV2 cells treated with Cuprizone. The arrows point to the mitochondria. (scale bars are 1μm and 50nm respectively).

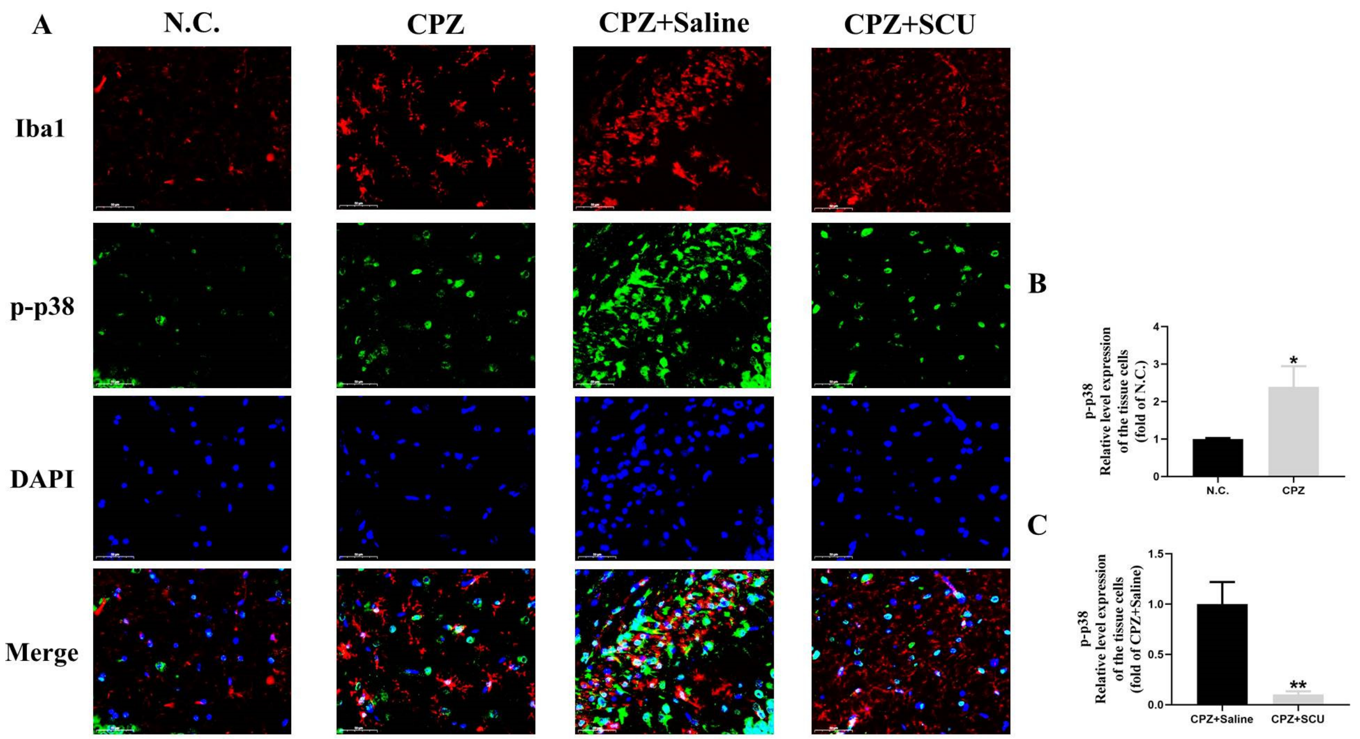

Figure S4. (A-C). Immunofluorescence observation of p-p38 fluorescence expression levels in brain tissue of mice after Scutellarin treatment. (zoom 36X, scale bars=50μm). * $p < 0.05$, ** $p < 0.01$. 3 mouse per group.
